# Oxygen Reserve Index as a Tool to Monitor Four Techniques of Oxygen Supplementation at Different Flow Rates in Dogs Sedated with Dexmedetomidine and an Opioid

**DOI:** 10.3390/ani13193077

**Published:** 2023-10-01

**Authors:** Luca Bellini, Giulia Maria De Benedictis

**Affiliations:** Department of Animal Medicine, Production and Health, University of Padova, Viale dell’Università 16, 35028 Legnaro, Italy; giuliamaria.debenedictis@unipd.it

**Keywords:** arterial partial pressure of oxygen, facemask, oxygen flow, oxygen reserve index, Venturi valve

## Abstract

**Simple Summary:**

Oxygen is a valuable resource, and its responsible use in veterinary medicine for treating dogs that require supplementation is of utmost importance. However, the effectiveness of various devices in enhancing arterial oxygen content during therapy remains inadequately explored. In patients breathing room air, pulse oximetry (SpO_2_) assesses arterial oxygen content based on its binding with hemoglobin, represented as a percentage. SpO_2_ is considered accurate in estimating arterial oxygenation for values between 100% and 80%. Physiologically, SpO_2_ is >98% when breathing room air and the device can also monitor the effectiveness of oxygen therapy. When the oxygen content in the blood rises above physiologic levels, the SpO_2_ remains constant. To address this, a new parameter, the oxygen reserve index, aids in estimating the animal’s arterial oxygen levels. Our study has determined that when compared with a vented face mask or flow-by methods, nasal prongs and Venturi valves, with the latter delivering a fixed oxygen amount, are the most effective methods for enriching inhaled oxygen flow. By employing these devices, coupled with the guidance of the oxygen reserve index, it becomes possible to optimize the minimum required oxygen flow rate for supplementation. This approach not only improves the efficacy of oxygen administration but also reduces wastage concurrently.

**Abstract:**

Respiratory dysfunction often decreases arterial oxygen content. Four common oxygen delivery techniques—flow-by, nasal prongs, a tight-vented mask, and a tight mask connected to a Venturi valve—were evaluated for their effectiveness in increasing the oxygen reserve index (ORi), a dimensionless index of oxygen content that provides additional information compared to traditional pulse oximetry (SpO_2_) during hyperoxia (PaO_2_ 100–200 mmHg), and that ranges from 0 to 1. Thirty-two dogs sedated with dexmedetomidine and an opioid were evenly divided into four groups based on the technique for oxygen administration. Each dog received oxygen at 1, 2, and 3 L/min by a single technique, and the amount of inhaled oxygen (FiO_2_) was measured at the level of the cervical trachea. At each flow rate, ORi and SpO_2_ were recorded. The flow-by method minimally increased the FiO_2_, and ORi reached its highest value only in 3 out of 8 dogs at the maximum flow rate. Other methods exhibited direct correlations between the oxygen flow rate and ORi (*p* < 0.001). These methods effectively increased FiO_2_ and ORi, with over half of the values exceeding 40% and 0.4, respectively. The tight-vented mask showed variable increases in FiO_2_, ranging between 22 and 90%. Despite method-dependent variations, all devices increased SpO_2_ > 98% as the FiO_2_ increased (*p* = 0.002). In conclusion, nasal prongs and the mask connected to the Venturi valve showed the highest correlation between the oxygen flow rate and the ORi. These results suggest that using these two techniques in conjunction with ORI can help in optimizing oxygen therapy.

## 1. Introduction

Hypoxemia or an arterial oxygen content lower than expected can occur in patients with diseases affecting the respiratory system or with respiratory muscle dysfunctions. Moreover, abnormal respiratory function during recovery from anesthesia or deep sedation can lead to a low arterial partial pressure of oxygen (PaO_2_) in dogs breathing room air [1,2]. Non or minimally invasive oxygen supplementation techniques are the first-line treatment to increase PaO_2_ and, therefore, oxygen tissue delivery. The veterinary literature reports several methods to enrich the inspired fraction of oxygen (FiO_2_), and the choice of one technique over the others depends on the severity of the respiratory failure, the patient tolerance, the peak inspiratory flow, and the desired FiO_2_ [2].

The nasal catheter is an effective method to administer oxygen, and an oxygen flow rate of 200 mL/kg/min increases the PaO_2_ above 350 mmHg in healthy dogs [2,3]. Its disadvantages include nasal bleeding during catheter insertion, dislodgement in agitated dogs, and the desiccation of nasal mucosa. Moreover, uncooperative dogs should be deeply sedated or anesthetized. Face masks, nasal prongs, or oxygen hoods are less invasive and are used to administer oxygen in noncritical patients. Those are variable performance devices because the delivered gas mixture has a variable FiO_2_ based on the patient’s peak inspiratory pressure that drives room air that mixes with oxygen. Most of the studies investigating the performances of those devices evaluated the maximal FiO_2_ achieved using a fixed oxygen flow rate [4,5]. Only one study quantified the PaO_2_ with an incremental oxygen flow rate delivered by nasal catheters [6].

Pulse oximetry estimates PaO_2_ indirectly by calculating the rate of light absorption at different wavelengths. PaO_2_ between 60 and 100 mmHg shows a linear correlation with peripheral hemoglobin oxygen saturation (SpO_2_); for this reason, this monitoring is a noninvasive, rapid, and easy method to guide oxygen therapy [7,8]. During hyperoxaemia, for PaO_2_ > 100 mmHg, the pulse oximeter displays a SpO_2_ > 98%, limiting its clinical relevance because it does not provide information on an impending decrease in PaO_2_ or whether the oxygen flow rate is causing severe hyperoxaemia with possible secondary atelectasis [9]. Moreover, the SpO_2_ showed limited reliability as a surrogate of the PaO_2_, underestimating the oxygen content in spontaneous breathing dogs inspiring room air [10].

The oxygen reserve index (ORi) is a dimensionless index measured by multiwavelength pulse co-oximetry, employing technology similar to traditional pulse oximetry. Arterial and venous blood hemoglobin exhibit distinct absorption properties at multiple wavelengths, and ORi estimates arterial oxygenation by combining the specific light absorption information for each form of hemoglobin. The manufacturer reports that within values of PaO_2_ between 100 and 200 mmHg, ORi readings range between 0.0 (no reserve) and 1.0 (full oxygen reserve) [11], although some studies demonstrate a statistically significant positive correlation for PaO_2_ slightly above 200 mmHg [12,13]. If no lung lesions or respiratory diseases are present, the theoretical value of FiO_2_ to obtain an ORi of one is 0.4 [11]. In a state of mild hyperoxia, the ORi overcomes the limitation of the traditional pulse oximeter and, in its sensing range, a direct correlation exists between this index and the PaO_2_ [12]. In anesthetized pediatric patients, ORi identified a decrease in PaO_2_ earlier than standard pulse oximetry [14]. Another study showed that ORi optimized oxygen titration to maintain a PaO_2_ around 100 mmHg in human patients recovering from breast surgery [15], indicating that this index can enhance oxygen therapy and avoid the unnecessary wastage of this gas. In veterinary medicine, the index was investigated in donkeys and dogs, showing a moderate correlation with the PaO_2_ measured by the blood gas analyzer [16,17]. Furthermore, ORi has a sensitivity of 87% in detecting a PaO_2_ > 150 mmHg when displaying a value > 0.45 in dogs [17].

This study aimed to evaluate if ORi was positively associated with an increase in FiO_2_, providing additional information on arterial oxygenation than SpO_2_, using four different methods for oxygen administration at three flow rates in sedated dogs classified by the American Society of Anesthesiologists as grade I and II. A secondary aim was to assess if there was a correlation between the oxygen flow rate and the ORi to predict the rise in oxygen content based on the flow administered.

## 2. Materials and Methods

### 2.1. Animals

The ethical committee of the University of Padova approved the study (OPBA 75/2021), and the owner of the dog gave written informed consent before any procedures. Thirty-two American Society of Anesthesiologists grade I and II dogs undergoing sedation for diagnostic or elective surgical procedures were enrolled in a randomized clinical study. The animals were judged healthy based on pre-anesthetic examination. Dogs younger than five months, pregnant, weighing less than 5 kg with a body condition score > 7 out of 9, or having a history of respiratory problems in the previous three weeks, were excluded. Dogs were fasted overnight with water withdrawn at 7.00 am. Hospital admission was in the morning at 9.00 am.

### 2.2. Experimental Protocol

Dogs admitted at the Veterinary Teaching Hospital were weighed and then received intramuscularly, in the quadriceps muscle, 4 µg/kg dexmedetomidine (Dexdomitor; Orion Corporation, Espoo, Finland) mixed with 0.15 mg/kg butorphanol (Dolorex; Animal Health Intervet Italia Srl, Segrate, Italy) or 0.15 mg/kg methadone (Semfortan, Dechra Veterinary Products Srl, Torino, Italy) if they underwent a diagnostic procedure or elective surgery, respectively. After 10 min, after the animal became recumbent, a cephalic vein was cannulated with an over-the-needle catheter (Delta Ven; Delta Med SPA, Viadana, Italy) of appropriate size. If the dog was not already in lateral recumbency, it was gently positioned laterally to maintain the side where the back legs were already placed, limiting stimulation and avoiding any additional pressure on the nondependent lung. Immediately after, a rigid urinary catheter (BUSTER Dog Catheter 10 Fr; KRUUSE, Langeskov, Denmark) with a Luer lock connection was inserted orally through the larynx and advanced until its tip reached the midpoint of the cervical trachea. The tracheal midpoint was defined as half the distance from the angle of the jaw to the shoulder joint. The length from the incisor to that point was premeasured, and a mark was made on the urinary catheter to identify the correct distance. To facilitate the catheter insertion, if sedation was light and the dog swallowed, propofol was administered intravenously to effect. The urinary catheter was connected to the sampling line of a side-stream capnography (sampling rate 200 mL/min) on a multiparameter monitor (Datex Ohmeda Monitor S/5; GE Healthcare, Chicago, IL, USA) and the presence of a standard capnogram waveform confirmed the correct positioning of the sampling catheter. A CO-oximeter (Rad-97™; Masimo Corp., Irvine, CA, USA) measured ORi and continuously displayed the plethysmogram and SpO_2_. According to the manufacturer’s instructions, the sensor probe (RD Rainbow Lite SET-1 NEO; Masimo Corp., Irvine, CA, USA) was wrapped around the tongue and then connected to the device to compensate for the background light. As instrumentation was completed, and at least 10 min after propofol administration, oxygen was administered with a bubbler (TR/200 oxygen therapy humidifier; flowmeter, Levate, Italy) connected to a flowmeter (RS flowmeter; flowmeter, Levate, Italy). Oxygen flow was administered in a fixed order at 1, 2, and 3 L/min using four different devices. In each dog, a single device was used, and subjects were evenly distributed in 4 groups by an online open-source randomizer (www.randomizer.org, accessed on 24 November 2021). Dogs in group F received flow-by oxygen through a 200 cm long tube (FIAB, Vicchio, Italy) placed 2 cm from the philtrum of the nose. In group P, oxygen was delivered with nasal prongs (FIAB, Vicchio, Italy) connected to the bubbler by a 160 cm long tube. Administration of oxygen was via a 210 cm long tube connected to a tight-vented face mask (Oxygen recovery mask; McCulloch Medical, Auckland, New Zealand) in group M and to a tight face mask (Oxygen Anaesthesia mask; Midmark Corporation, Dayton, OH, USA) connected to a Venturi valve (Venturi Barrel; Flexicare medical limited, Mountain Ash, UK) in group V. The Venturi valve supplied a fixed FiO_2_ of 40%.

### 2.3. Measurements

The clinical information recorded in each dog was weight, body condition score, age, and recumbency. A multiparameter monitor (Datex Ohmeda Monitor S/5; GE Healthcare, Chicago, IL, USA) measured the FiO_2_ and the end-tidal carbon dioxide partial pressure (EtCO_2_) via a sidestream analyzer connected to the tracheal catheter by a 300 cm long non-compliant tube (Gas sampling line; GE Healthcare, Helsinki, Finland). Noninvasive arterial blood pressure was measured with an oscillometric monitor (PetTrust Blood Pressure Monitor, BioCare, Aster Electrical Co. Ltd., Taoyuan City, Taiwan) with the cuff placed on the upper metatarsal region and its width selected by a ruler provided by the manufacturer. The CO-oximeter measured SpO_2_, ORi, and the pulse rate (PR), displayed the plethysmogram, and was connected to the tongue sensor. RR was obtained by visually counting the chest movement over one minute. Before starting the measurements, each flow rate was maintained for at least 3 min to equilibrate the FiO_2_. The baseline measurements were recorded immediately before oxygen administration. Rectal temperature was measured with a digital thermometer (Digi-Vet SC 12, KRUUSE, Langeskov, Denmark) at the end of the last recording.

### 2.4. Statistical Analysis

All statistical analyses were performed with an open-source statistical package (R, The R Foundation for Statistical Computing, Vienna, Austria). Continuous variables were assessed for normal distribution using the Shapiro–Wilk test. If symmetry was confirmed, data were expressed as mean ± standard deviation while non-normally distributed variables were reported as median (minimum–maximum). The Fisher’s exact test compared the proportions of ordinal variables among groups. One-way ANOVA or the Kruskal–Wallis rank-sum test was used to compare weight, body condition score, age, and temperature among groups. An analysis of covariance (ANCOVA) was employed to compare FiO_2_, ORi, SpO_2_, and EtCO_2_ among groups, with the method used to deliver oxygen, the oxygen flow rate, and their interaction inserted in the model as fixed effects. Alternatively, a Kruskal–Wallis rank-sum test was used for nonparametric analysis of variance when appropriate. A Tukey post hoc pairwise comparison test was used if statistical differences were present. The same analysis was performed to detect differences in SpO_2_ and ORi, considering recumbency, the type of opioid used, the technique of oxygen administration, and their interactions as fixed effects. In all groups, ORi values were plotted against the flow rate expressed as L/kg/min and analyzed using linear regression. The regression line was forced to start from the origin, and a range of flow values between 0 and 0.5 L/kg/min was considered to identify an equation that could predict the oxygen flow rate based on weight to achieve a specific value of ORi. A value of *p* < 0.05 was considered statistically significant.

## 3. Results

All dogs completed this study without complications. Table 1 reports the demographic data of the animals. Dogs in group M had a statistically lower body weight than those in group V (*p* = 0.045). The cardiovascular and respiratory variables showed no significant differences among the groups for each flow rate (Table 2). Propofol was administered to all dogs at 1 to 1.5 mg/kg.

**Figure 1:**
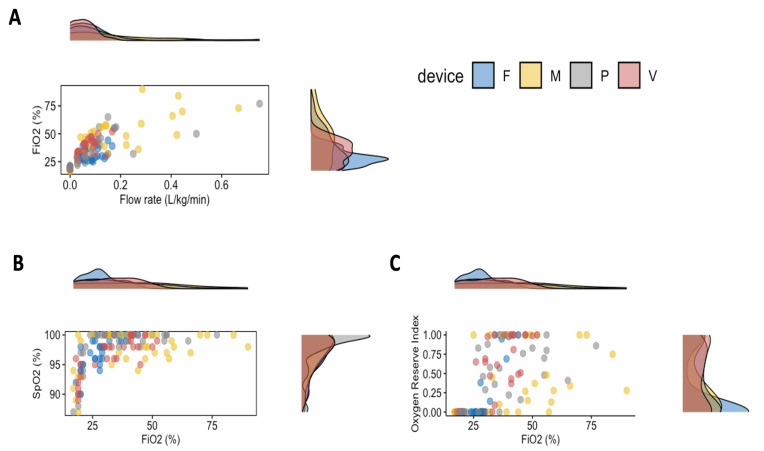
Dot plot and paired density plot of (**A**) the inspired fraction of oxygen (FiO_2_) achieved for each device at any flow rate; (**B**) value of SpO_2_ over FiO_2_; (**C**) value of ORi over FiO_2_. The following administering methods were used: a cannula placed 2 cm from the nostril (F); nasal prongs (P); a tight-vented mask (M); or a tight mask connected to a Venturi valve (V) delivering a fixed oxygen fraction of 40%.

The oxygen flow rate, the method of oxygen delivery, and the interaction of those factors had an effect on the FiO_2_ (Table 3). Figure 1 and Table 4 indicate that several dogs received a flow rate below 0.2 L/kg/min. Regardless of the administration method, increasing the oxygen flow rate proportionally increased the FiO_2_ except for the flow-by group, which showed a minimal rise (Figure 1A). Both ORi and SpO_2_ increased proportionally to the FiO_2_ (Figure 1 B,C).

The method used to deliver oxygen influenced the EtCO_2_ (*p* < 0.001) and the ORi (*p* = 0.024). Specifically, EtCO_2_ was statistically lower in group M than in groups F (*p* < 0.001) and P (*p* < 0.001) and lower in group V than in group P. The ORi was lower in group F compared to the other groups (*p* = 0.012). The FiO_2_ was significantly associated with the increase in the SpO_2_ (*p* < 0.001), although the device used to deliver the oxygen showed no effects (Table 3 and Table 4, Figure 1B).

In group F, 4 out of 8 (50%) dogs had an ORi of zero regardless of the flow used, and only 3 dogs reached an ORi value of one with an oxygen flow rate of 3 L/min. Regardless of the flow rate, 5 out of 8 dogs included in group F exhibited an ORi < 0.5. In the other groups, an ORi > 0.5 was reached by 50% of the dogs at an oxygen flow rate of 2 L/minute, and, in groups M and group V, five dogs in each group reached an ORi of one with an oxygen flow rate of 3 L/min.

**Figure 2:**
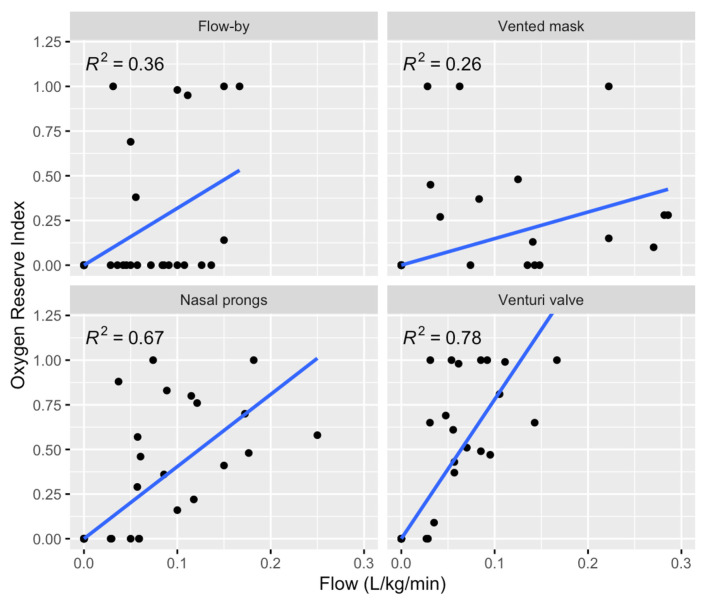
Dot plot of oxygen flow rate over ORi with the result of the linear regression model. The following administering methods were used: a cannula placed 2 cm from the nostril (F); nasal prongs (P); a tight-vented mask (M); or a tight mask connected to a Venturi valve (V) delivering a fixed oxygen fraction of 40%.

The linear regression between the oxygen flow rate over body weight and the ORi indicated a good correlation in the four methods (Figure 2, Table 5). The best correlation was observed with the Venturi valve, with an r^2^ of 0.78 (*p* < 0.001).

## 4. Discussion

This study identified a positive correlation between the oxygen flow rate, the FiO_2_, and the increases in ORi and SpO_2_. The flow-by was the less effective administration method to enrich the inspired gas with oxygen. No statistical differences appeared among the other methods regarding the values of ORi reached over different oxygen flow rates. The tight face mask with a Venturi valve rose the ORi more rapidly and efficiently than the other methods.

The oxygen flow rates used in our study were those commonly administered in clinical settings [2]. Moreover, the administration of oxygen by flow-by, nasal catheter, or face masks causes a rapid rise of FiO_2_ until 3 L/min, and, above that rate, the increment slows, and it reaches a plateau [4,5]. To avoid the unnecessary waste of oxygen, we decided to maintain the rate below that value. Normalizing the oxygen flow for the weight of the animals, most of the dogs received a flow of oxygen between 0.03 and 0.2 L/kg/min, which is considered a clinically acceptable flow that optimizes the oxygen administration, avoiding discomfort due to the unpleasant sensation caused by turbulent gas flow [3].

Peripheral SpO_2_ is a simple and continuous method of monitoring patients receiving oxygen therapy, and, in our study, FiO_2_ affected the hemoglobin oxygen saturation. On the other hand, the administration method to deliver oxygen did not affect the value of SpO_2_, while it significantly influenced the increase in ORi. This result reflects the ability of this index to estimate the arterial oxygen content for a wider range of PaO_2_ than SpO_2_ [11]. Monitoring hyperoxia could minimize absorption atelectasis, limiting a further collapse of the lung caused by sedation [9], and, in human medicine, ORi was successfully used to titrate oxygen therapy for maintaining a target PaO_2_ close to 100 mmHg [15]. The ORi was successfully used to optimize preoxygenation before anesthesia induction because of its ability to detect an impending decrease in PaO_2_ before traditional pulse oximetry in humans [11].

Oxygen delivered by flow-by caused a rise in the SpO_2_ but led to a limited increase in the ORi. Moreover, increasing the oxygen flow did not produce a clinically relevant rise in the FiO_2_, suggesting that most of the flow is wasted in the surrounding environment rather than being inhaled, in agreement with another study that administered oxygen by flow-by [5]. Ambros et al. (2018) [18], using a flow-by technique with 0.1 L/kg/min for 3 min, reached a mean FiO_2_ of 0.30, in agreement with our results. In different studies, the PaO_2_ achieved with 0.1 L/kg/min flow-by oxygen was variable [6]. Similarly, flow-by increased SpO_2_, although ORi exhibited variable rises, with values of one observed in only three dogs at a high oxygen flow rate. Additionally, half of the dogs showed an ORi of 0.0 regardless of the oxygen flow rate.

All the other methods for oxygen administration increased ORi proportionally to the oxygen flow rate in almost all the dogs. The administration of oxygen by human-designed nasal prongs is common in veterinary medicine. Our results showed an almost direct correlation between the oxygen flow rate and the FiO_2_, as reported in a previous study with similar findings using bilateral nasal catheters [3]. The volume of the nasal cavity, the oropharynx, and the nasopharynx represent an oxygen reservoir. The oxygen fills that space proportionally to the flow provided during the expiratory pause. Our study identified a direct FiO_2_-dependent increase in ORi with nasal prongs. Despite values of FiO_2_ above 0.4, ORi measurements were lower than expected in several dogs, suggesting a worsening of respiratory function due to the recumbency and the use of sedatives in addition to the detrimental effects of absorption atelectasis associated with elevated FiO_2_ [9].

Oxygen face masks are variable performance devices, and the cone generally has a rubber diaphragm to minimize oxygen leakage once in place on the muzzle. During the expiratory pause, oxygen fills the volume inside the mask as it flows from the flowmeter. The amount of oxygen inhaled depends on the respiratory rate and the amount of space between the mask and the muzzle of the dog acting as a reservoir [1]. The reservoir volume is variable, and, to match the peak inspiratory flow, masks frequently have side vented holes for room air entrance, leading to a variable oxygen dilution with room air. The dilution may explain the flow rate variability in FiO_2_ and ORi observed in our study. Moreover, the FiO_2_ seems to stabilize as the flow rate rose because less entrainment of room air occurred to meet peak inspiratory flow, as previously observed [5]. Because we used masks with side holes, it is likely that oxygen was vented as the flow increased. In this current study, severe hypercapnia was not observed, indicating that rebreathing, if present, was minimal.

A Venturi valve can be connected to a face mask and converts the device into an oxygen supplementation fixed performance device. That device mixes air and oxygen at a specific and fixed ratio, regardless of the flow of oxygen administered, as long as the total flow rate matches the peak inspiratory flow of the patient. The manufacturer produces different valves, each mixing gases to achieve a constant FiO_2_. For this study, we used a valve delivering a FiO_2_ of 0.4 because the estimated arterial partial pressure of oxygen is 200 mmHg, representing the upper limit of the sensing range of ORi [11]. In our study, the FiO_2_ measured with a flow rate of 1 L/min was less than 0.40 in most dogs, suggesting that the peak inspiratory flow was not matched. As the flow increased, the inhaled oxygen fraction rose until it reached a plateau matching the FiO_2_ of the selected valve connector and the value of ORi matched the FiO_2_ [11,14].

For each oxygen administration technique, an equation was derived to calculate the minimum flow required to achieve a specific ORi value. The objective was to provide a tool for optimizing oxygen therapy based on the desired PaO_2_. ORi has been used for oxygen therapy optimization in human medicine, with the aim of avoiding excessive oxygen flows [15]. Among the techniques evaluated, the correlation was poor in group F, likely because most of the ORi values remained at zero despite an increase in the oxygen flow rate. In dogs with a unilateral nasal catheter, a flow rate of 0.1 L/kg/min provides a PaO_2_ of approximately 200 mmHg [3], which is within the ORi sensing range. However, apart from that study, there were no similar investigations exploring alternative oxygen delivery techniques.

Some dogs developed mild hypercapnia, likely as a result of the adverse effects of the combination of dexmedetomidine with opioids [19]. Because atelectasis may develop during sedation, an additional impairment in the gas exchange may have occurred [9,20]. Although dogs receiving oxygen with a tight-vented face mask appeared to have lower EtCO_2_ levels than the other groups, this result may be an incidental finding, as this study did not specifically aim to detect differences in that variable. Bradycardia is frequently associated with dexmedetomidine, and the pulse rates measured in this study are similar to those reported in the literature, especially when opioids are coadministered [21].

The effect of positioning may represent a limitation in this study, and it may explain the lower estimated arterial oxygen content recorded in some dogs, with values of ORi not matching the FiO_2_. In lateral recumbency, atelectasis develops, and the lung functional residual capacity decreases with intrapulmonary shunt development, and sedatives worsen this condition [9,20]. Also, in laterally recumbent anesthetized donkeys, the matching of PaO_2_ and ORi had poor results in some animals, suggesting an effect of positioning [16]. Moreover, ORi rises proportionally until venous hemoglobin saturation reaches a plateau, and a value of one estimates a PaO_2_ ≥ 200 mmHg; this could have underestimated the differences among the methods. Alpha-2 agonists cause peripheral vasoconstriction with abnormal oxygen extraction [19]. This condition may alter the venous hemoglobin saturation, representing an additional factor that could have affected ORi measurements. In human medicine, a positive correlation between ORi and PaO_2_ has been demonstrated up to values of 240 mmHg [12,13]. However, in dogs, this condition has not been studied yet, and there could be an underestimation of PaO_2_ by ORi based on the measured FiO_2_.

Another limitation of this study is the difference in recumbency and the use of either butorphanol or methadone in association with dexmedetomidine. Although the ANCOVA did not show any statistically significant effects of recumbency on the values of ORi and SpO_2_, Barletta et al. reported that in lateral recumbency, the pulmonary pattern suggestive of atelectasis, as evaluated by radiography, was more pronounced in right lateral recumbent dogs compared to other recumbent positions [9]. In this current study, even if a different lung pattern could be present clinically, respiratory function was preserved. Methadone can induce panting when used alone, but its combination with medetomidine attenuates this effect [22]. Furthermore, in a comparative study, no differences in respiratory rates were detected in dogs receiving intravenous medetomidine with either methadone or butorphanol [23]. In this current study, the alpha-2 agonist’s effects may have mitigated the difference in the respiratory rate of both opioids, as indicated by the absence of statistical effects of the opioid used on SpO_2_ or ORi. Lastly, the effect of tidal volume was not considered in this study and may have limited the rise in the FiO_2_ measured. Dexmedetomidine increases the tidal volume that is negatively associated with the FiO_2_, achieved by maintaining a constant oxygen insufflation rate [24,25].

## 5. Conclusions

Oxygen administration via each of the studied devices increased SpO_2_, and ORi exhibited a positive correlation with proportional increases in FiO_2_. The increase in ORi was pronounced in all techniques but the flow-by method. The nasal prongs and Venturi valve demonstrated the strongest correlation between the ORi values and oxygen flow rates, expressed as L/kg/min. The use of these two techniques in conjunction with ORi may help to prevent wastage and optimize oxygen therapy.

## Figures and Tables

**Table 1:** Demographic data, body temperature, the number of animals placed in left or right lateral recumbency, and number of animals that received dexmedetomidine and methadone or butorphanol of the four groups. Dogs were grouped based on the methods used to deliver oxygen via a bubbler. The following administering methods were used: a cannula placed 2 cm from the nostril (flow-by); nasal prongs; a tight-vented mask; or a tight mask connected to a Venturi valve (Venturi) delivering a fixed oxygen fraction of 40%. Data are reported as mean ± standard deviation or median (minimum–maximum).

	Flow-by	Nasal Prongs	Vented Mask	Venturi	*p*-Value
Weight (kg)	24.8 ± 6.2	21.3 ± 10.2	16.4 ± 12.4	30.0 ± 7.0 *	0.045
BCS	5 (4–6)	6 (5–7)	5 (3–6)	5 (4–6)	0.222
Age (months)	13 (5–97)	80 (18–149)	50 (10–180)	24 (7–98)	0.055
Sex (m:f)	4:4	5:3	5:3	2:6	0.392
Temperature (°C)	38.0 ± 1.0	37.6 ± 1.1	37.7 ± 1.0	38.6 ± 0.6	0.158
Lateral recumbency (left:right)	7:1	5:2	2:6	4:4	0.067
Methadone vs butorphanol	3:5	3:5	7:1	2:6	0.060

BCS, body condition score; f, female; m, male; * statistically significant difference (*p* < 0.05) between vented mask and Venturi.

**Table 2:** Respiratory and cardiovascular variables in dogs sedated with dexmedetomidine and butorphanol or methadone. Animals were grouped based on the methods used to deliver oxygen via a bubbler: a cannula placed 2 cm from the nostril (flow-by); nasal prongs; a tight-vented mask; or a tight mask connected to a Venturi valve (Venturi) delivering a fixed oxygen fraction of 40%. Data are reported as mean ± standard deviation or median (min–max).

	EtCO_2_ (mmHg)	RR (Breath/min)	PR (Beat/min)	MAP (mmHg)
Flow-by	52 ± 5	15 (7–27)	60 ± 21	92 ± 13
Vented mask	46 ± 4	14 (5–50)	56 ± 14	93 ± 16
Nasal prongs	52 ± 5	14 (6–37)	48 ± 13	91 ± 21
Venturi	49 ± 6	15 (6–49)	64 ± 20	95 ± 12

EtCO_2_, end-tidal carbon dioxide partial pressure; MAP, mean systemic arterial pressure; PR, pulse rate; RR, respiratory rate.

**Table 3:** Results of the ANCOVA (F-stat) in dogs sedated with dexmedetomidine and butorphanol or methadone. The analysis investigates: (1) the effects, on the inspired fraction of oxygen (FiO_2_), of different flow rates of oxygen or the method used to deliver the oxygen (method), and their interaction; (2) the effects, on EtCO_2_, SpO_2_, and ORi, of the inspired fraction of oxygen or the method used to deliver the oxygen (method), and their interaction; and (3) the effect, on SpO_2_ and ORi, of the opioid used or the recumbency assumed during this study, the method used to deliver the oxygen (method), and their interaction.

	Flow rate		Method		Flow rate × Method	
	*F*-stat	*p*-value	*F*-stat	*p*-value	*F*-stat	*p*-value
FiO_2_	162.2	<0.001	13.5	<0.001	3.1	0.030
	FiO_2_		Method		FiO_2_ × Method	
	*F*-stat	*p*-value	*F*-stat	*p*-value	*F*-stat	*p*-value
EtCO_2_	2.0	0.157	13.0	<0.001	3.1	0.030
SpO_2_	59.9	<0.001	0.9	0.424	5.5	0.002
ORi	70.9	<0.001	3.3	0.024	11.8	<0.001
	Opioid		Method		Opioid × Method	
	*F*-stat	*p*-value	*F*-stat	*p*-value	*F*-stat	*p*-value
SpO_2_	1.9	0.171	0.6	0.616	1.1	0.352
ORi	2.1	0.150	1.8	0.143	1.9	0.127
	Recumbency		Method		Recumbency × Method	
	*F*-stat	*p*-value	*F*-stat	*p*-value	*F*-stat	*p*-value
SpO_2_	1.1	0.287	0.4	0.776	0.1	0.953
ORi	0.9	0.357	1.8	0.154	0.5	0.654

EtCO_2_, end-tidal carbon dioxide partial pressure; ORi, oxygen reserve index.

**Table 4:** Flow rate of oxygen administered and inspired fraction of oxygen (FiO_2_) attained in dogs sedated with dexmedetomidine and butorphanol or methadone. The dogs were grouped based on the methods used to deliver oxygen: a cannula placed 2 cm from the nostril (flow-by); nasal prongs; a tightly vented mask; or a tightly fitting mask connected to a Venturi valve (Venturi) delivering a fixed oxygen fraction of 40%. Data are reported as mean ± standard deviation or median (minimum–maximum).

	Flow Rate (L/kg/min)	FiO_2_ (%)
Flow-by		
0 L/min	-	19.8 ± 0.9
1 L/min	0.04 (0.03–0.06)	29.5 ± 4.8
2 L/min	0.09 (0.06–0.11)	30.9 ± 5.9
3 L/min	0.13 (0.09–0.17)	33.9 ± 6.8
Vented mask		
0 L/min	-	18.6 ± 1.2
1 L/min	0.11 (0.03–0.22)	43.9 ± 11.3
2 L/min	0.21 (0.05–0.44)	52.1 ± 20.4
3 L/min	0.31 (0.08–0.66)	57.1 ± 16.2
Nasal prongs		
0 L/min	-	20.0 ± 1.4
1 L/min	0.05 (0.03–0.24)	30.4 ± 4.6
2 L/min	0.11 (0.05–0.50)	41.0 ± 10.5
3 L/min	0.16 (0.09–0.75)	51.6 ± 16.2
Venturi		
0 L/min	-	19.2 ± 0.7
1 L/min	0.03 (0.03–0.06)	31.2 ± 3.2
2 L/min	0.06 (0.05–0.11)	40.2 ± 6.3
3 L/min	0.09 (0.08–0.17)	42.8 ± 6.7

FiO_2_, inspired fraction of oxygen.

**Table 5:** Linear regression analysis results for the dogs that received oxygen delivered by 4 methods: flow-by, nasal prongs, via a tight-vented mask, or a tight mask with a Venturi valve that delivers a fixed fraction of oxygen of 40%.

Administration Methods	r^2^	Derived Equation	*p*-Value
Flow-by	0.36	ORi = 3.18 × Flow rate	<0.001
Vented mask	0.26	ORi = 1.49 × Flow rate	0.001
Nasal prongs	0.67	ORi = 4.04 × Flow rate	<0.001
Venturi	0.78	ORi = 7.81 × Flow rate	<0.001

## Data Availability

The data presented in this study are available on request from the corresponding author upon reasonable request.
